# Letter to the Editor – Brazilian and US-American Researchers Cite Articles Written by Brazilian Authors in Revista Brasileira de Ortopedia

**DOI:** 10.1055/s-0044-1785468

**Published:** 2024-04-10

**Authors:** Konradin Metze, Rosana C. Morandin-Reis

**Affiliations:** 1 Curso de Pós-Graduação em Fisiopatologia Médica, Faculdade de Ciências Médicas, Universidade Estadual de Campinas, Campinas, SP, Brasil; 2 Faculdade de Ciências Médicas, Universidade Estadual de Campinas, Campinas, SP, Brasil

In a previous work Giordano et al. 1 studied the frequency of citations to articles previously published in RBO. They had focused on the reference lists of the last 15 articles published in 2020 in RBO and 5 international orthopedic journals. They found 10 citations among 386 papers (2.6%) of the journal, but could not find any citation to RBO in the international journals. So the authors concluded that Brazilians do not cite Brazilians.

We re-analyzed this issue using a considerably larger database and two different statistical approaches:

We based our study on Clarivate's Data which contains bibliographic information and citations of more than 20,000 indexed scientific journals. We restricted all searches to original articles, reviews and case reports. For the first mathematical evaluation we looked for all RBO articles published between 2007 and 2020 and citations to them in 2020-2021. There were 1,318 publications written by Brazilian authors with 794 citations to them in 2020 (with 34 self-citations). Among these citations, 163 (20.7%; CI95%: 17.9%-23.7%) had Brazilian, 176 (22.0%; CI95%: 19.5%-25.4%) had US-American, and 106 (13.4%; CI95%: 11.1%-16,0%) had Chinese authors or co-authors. The confidence intervals suggested that Brazilians and US-Americans cited RBO articles of Brazilian origin with a similar frequency. 2

In the second approach we collected separately for each year between 2013 and 2020 RBO articles written by Brazilian authors ( Table 1 ). Then, we looked for the citing articles which had been published in the same year or the following biennium (now called three-years-period) and counted separately the number of articles written by Brazilian, US-American or Chinese authors/coauthors.

**Table 1:** Number of publications and citations in *RBO*

Year	all articles a	articles of Brazilian authors b	3 years citations to Brazilians c	Mean Nr. of all citing articles d	Mean Nr of citing articles of Brazilians e	Mean Nr of citing articles by US-Americans f	Mean Nr of citing articles by Chinese g
2013	98	93	17	0.183	0.097	0.032	0.022
2014	119	110	54	0.491	0.209	0.055	0.064
2015	125	118	101	0.856	0.263	0.127	0.119
2016	122	115	141	1.226	0.270	0.374	0.148
2017	130	112	163	1.455	0.241	0.473	0.116
2018	125	101	165	1.634	0.267	0.356	0.277
2019	121	104	125	1.202	0.240	0.231	0.183
2020	131	109	123	1.128	0.248	0211	0.193
mean ± st. dev	121.38 ± 10.2	107.75 ± 8.10	111.13 ± 52.16	1.022 ± 0.487	0.229 ± 0.057	0.232 ± 0.159	0.140 ± 0.079

a all articles, reviews and case reports, with authors of all nationalities published in this year in RBO.

b articles, reviews and case reports written by Brazilian authors in this year in RBO.

c total number of citing articles related to Brazilian publications in the three-years-period.

d mean number of all articles (three-years-period) citing publications written by Brazilian authors.

e mean number of citing articles (three-years-period) written by Brazilian researchers to Brazilian publications.

f mean number of citing articles (three-years-period) written by US-American researchers to Brazilian publications.

g mean number of citing articles (three-years-period) written by Chinese researchers to Brazilian publications.

A total of 971 documents were included, and 862 had been written by Brazilian researchers. These publications yielded a total of 889 citations. Self-citations were not found. Then we calculated for the Brazilian RBO publications, individually for each year, the mean number of citing articles written by Brazilian, US-American or Chinese researchers. The global Friedman exact test for dependent groups showed significant differences (p = 0.030). In order to detect differences between individual groups in a post-hoc procedure, we applied the Wilcoxon exact test followed by an adjustment of the alpha-level, which was necessary due to multiple testing. 3 We could not demonstrate a significant difference between mean citations to Brazilian documents coming from Brazilian or the US-American authors (p = 0.945). But there were fewer citations made by Chinese researchers, when compared with Brazilian (p = 0.016) or US-American authors (p = 0.023). The alpha-level was adjusted to α = 0.025 did not change these results.

All statistical procedures were performed with SPSS 22 software.

Our two statistical investigations showed that documents published in RBO by Brazilian authors had been cited by Brazilian and US-American researchers to an equal amount and to some lesser extent by Chinese authors and researchers from other countries. This suggests that Brazilian and US-American authors take Brazilian orthopedic research into consideration in a similar way. Since our study was performed on a very large international database, we believe that our results are highly representative of the reality.
