# Supplementary material for: Letter to the Editor – Brazilian and US-American Researchers Cite Articles Written by Brazilian Authors in Revista Brasileira de Ortopedia
Source: Rev Bras Ortop (Sao Paulo). 2024 Apr 10;59(2):e331–3. doi: 10.1055/s-0044-1785468 (PMC11006513; doi:10.1055/s-0044-1785468)
Supplement: Related texts

# Research article: Carta ao editor – Pesquisadores brasileiros e norte-americanos citam artigos escritos por autores brasileiros na Revista Brasileira de Ortopedia

Em trabalho anterior, Giordano et al. 1 estudaram a frequência de citações de artigos previamente publicados na RBO. Eles se concentraram nas listas de referências dos últimos 15 artigos publicados em 2020 na RBO e em 5 revistas ortopédicas internacionais. Eles encontraram 10 citações entre 386 artigos (2,6%) da revista, mas não encontraram nenhuma citação à RBO nas revistas internacionais. Então os autores concluíram que os brasileiros não citam brasileiros.

Reanalisamos essa questão usando um banco de dados consideravelmente maior e duas abordagens estatísticas diferentes:

Baseamos nosso estudo nos dados da Clarivate que contém informações bibliográficas e citações de mais de 20.000 revistas científicas indexadas. Restringimos todas as pesquisas a artigos originais, revisões e relatos de casos. Para a primeira avaliação matemática procuramos todos os artigos da RBO publicados entre 2007 e 2020 e citações dos mesmos em 2020-2021. Foram 1.318 publicações de autores brasileiros com 794 citações em 2020 (com 34 autocitações). Dentre essas citações, 163 (20,7%; IC95%: 17,9%-23,7%) tiveram autores brasileiros, 176 (22,0%; IC95%: 19,5%-25,4%) tiveram autores norte-americanos e 106 (13,4%; IC95%: 11,1). %-16,0%) tiveram autores ou coautores chineses. Os intervalos de confiança sugeriram que brasileiros e norte-americanos citaram artigos da RBO de origem brasileira com frequência semelhante. 2

Na segunda abordagem coletamos separadamente para cada ano entre 2013 e 2020 artigos da RBO escritos por autores brasileiros ( Tabela 1 ). Em seguida, procuramos os artigos citantes publicados no mesmo ano ou no biênio seguinte (agora denominado período de três anos) e contamos separadamente o número de artigos escritos por autores/coautores brasileiros, norte-americanos ou chineses.

**Tabela 1:** Número de publicações e citações na RBO

Ano	Todos os artigos a	Artigos de autores brasileiros b	Citações de 3 anos para brasileiros c	Número médio de todos os artigos que citaram d	Média de artigos citados por brasileiros e	Número médio de citações de artigos de norte-americanos f	Número médio de artigos citados por chineses g
2013	98	93	17	0.183	0.097	0.032	0.022
2014	119	110	54	0.491	0.209	0.055	0.064
2015	125	118	101	0.856	0.263	0.127	0.119
2016	122	115	141	1.226	0.270	0.374	0.148
2017	130	112	163	1.455	0.241	0.473	0.116
2018	125	101	165	1.634	0.267	0.356	0.277
2019	121	104	125	1.202	0.240	0.231	0.183
2020	131	109	123	1.128	0.248	0211	0.193
média ± desvio padrão	121.38 ± 10.2	107.75 ± 8.10	111.13 ± 52.16	1.022 ± 0.487	0.229 ± 0.057	0.232 ± 0.159	0.140 ± 0.079

a todos os artigos, revisões e relatos de casos, com autores de todas as nacionalidades publicados neste ano na RBO.

b artigos, revisões e relatos de casos escritos por autores brasileiros neste ano na RBO.

c número total de artigos citados relacionados a publicações brasileiras no triênio.

d número médio de todos os artigos (período de três anos) que citam publicações de autores brasileiros.

e número médio de artigos citados (período de três anos) escritos por pesquisadores brasileiros para publicações brasileiras.

f número médio de artigos citados (período de três anos) escritos por pesquisadores norte-americanos para publicações brasileiras.

g número médio de artigos citados (período de três anos) escritos por pesquisadores chineses para publicações brasileiras.

Foram incluídos 971 documentos, sendo 862 de autoria de pesquisadores brasileiros. Essas publicações renderam um total de 889 citações. Não foram encontradas autocitações. Em seguida, calculamos para as publicações brasileiras da RBO, individualmente para cada ano, o número médio de artigos citados escritos por pesquisadores brasileiros, norte-americanos ou chineses. O teste exato de Friedman para grupos dependentes apresentou diferenças significativas (p = 0,030). Para detectar diferenças entre os grupos individuais em um procedimento post-hoc, aplicamos o teste exato de Wilcoxon seguido de um ajuste do nível alfa, que foi necessário devido a múltiplos testes. 3 Não foi possível demonstrar diferença significativa entre as médias de citações de documentos brasileiros provenientes de autores brasileiros ou norte-americanos (p = 0,945). Mas houve menos citações feitas por pesquisadores chineses, quando comparados com autores brasileiros (p = 0,016) ou norte-americanos (p = 0,023). O nível alfa foi ajustado para α = 0,025, não alterando esses resultados.

Todos os procedimentos estatísticos foram realizados com o software SPSS 22.

Nossas duas investigações estatísticas mostraram que documentos publicados na RBO por autores brasileiros foram citados por pesquisadores brasileiros e norte-americanos em igual quantidade e, em menor grau, por autores chineses e pesquisadores de outros países. Isso sugere que autores brasileiros e norte-americanos levam em consideração a pesquisa ortopédica brasileira de maneira semelhante. Como o nosso estudo foi realizado numa base de dados internacional muito grande, acreditamos que os nossos resultados são altamente representativos da realidade.
